# An analysis of clinical risk factors for adolescent scoliosis caused by spinal cord abnormalities in China: proposal for a selective whole-spine MRI examination scheme

**DOI:** 10.1186/s12891-020-3182-z

**Published:** 2020-03-24

**Authors:** Wei Xu, Xiangyang Zhang, Ying Zhu, Xiaodong Zhu, Zhikun Li, Dachuan Li, Jianjun Jia, Liwei Chen, Silian Wang, Yushu Bai, Ming Li

**Affiliations:** 1 Department of Orthopedics, Tongren Hospital, Shanghai Jiao Tong University School of Medicine, 1111 XianXia Road, Shanghai, 200336 People’s Republic of China; 2 Department of Radiology, Tongren Hospital, Shanghai Jiao Tong University School of Medicine, 1111 XianXia Road, Shanghai, 200336 People’s Republic of China; 3 NO.7 College team, PLA Naval Medical University, 800 Xiangyin Road, Shanghai, 200443 People’s Republic of China; 4 Department of Spine, Shanghai Changhai Hospital, PLA Naval Medical University, 168 Changhai Road, Shanghai, 200433 People’s Republic of China

**Keywords:** Adolescent scoliosis, MRI, Neural axis abnormalities, Prevalence

## Abstract

**Background:**

Approximately 80% of adolescent scoliosis cases are idiopathic, and some non-idiopathic scoliosis cases caused by spinal cord abnormalities are misdiagnosed as idiopathic scoliosis. This study examined the risk factors for non-idiopathic scoliosis with intramedullary abnormalities, explored the feasibility of whole-spine MRI, and provided a theoretical basis for the routine diagnosis and treatment of adolescent idiopathic scoliosis.

**Method:**

The clinical data of adolescent scoliosis patients who were admitted to Shanghai Tongren Hospital and Shanghai Changhai Hospital between July 1, 2013, and December 31, 2018, were reviewed. According to the whole-spine MRI results, the patients were divided into either the idiopathic group or the intramedullary abnormality group. Sex, age, main curvature angle, main curvature direction, kyphosis angle, scoliosis type, coronal plane balance, sagittal plane balance, abdominal wall reflex, sensory abnormality, ankle clonus and tendon reflexes were compared between the two groups. Student’s t test was used to evaluate the differences in the continuous variables, and the chi-square test was used to evaluate the differences in the categorical variables. Fisher’s exact test was applied to detect the difference in the rate of intraspinal anomalies between the groups. Logistic regression was used to evaluate the correlation between the multivariate risk factors and intramedullary abnormalities.

**Result:**

A total of 714 adolescent scoliosis patients with a mean age of 13.5 (10–18 years) were included in the study, and intramedullary abnormalities were found in 68 (9.5%) patients. There were statistically significant differences in the incidence rates of intramedullary abnormalities between males and females, left and right thoracic curvatures, angular scoliosis and smooth scoliosis, and abnormal abdominal wall reflex and ankle clonus (*P* < 0.01). Logistic regression showed that the ratios for sex, scoliosis direction, scoliosis type, abdominal wall reflex and ankle clonus were 2.987, 3.493, 4.823, 3.94 and 8.083, respectively. The ROC curve showed a sensitivity of 66.18% and a specificity of 89.01%, and the Youden index corresponding to the optimal critical point was 0.5519.

**Conclusion:**

Risk factors associated with adolescent scoliosis caused by abnormal intramedullary abnormalities included male sex, thoracic scoliosis on the left side, sharp curvature of the spine, abnormal abdominal wall reflex and ankle clonus. In adolescent scoliosis patients, the incidence of scoliosis caused by intramedullary abnormalities was approximately 9.5%. These clinical indicators suggest that there is a high-risk adolescent scoliosis population who should undergo whole-spinal MRI preoperatively to rule out intramedullary abnormalities.

## Background

Scoliosis is a common three-dimensional spinal deformity that can be clearly diagnosed with a physical examination. Scoliosis can be classified as idiopathic, congenital, or neuromuscular scoliosis; neurofibromatosis scoliosis; spinal scoliosis caused by intramedullary abnormalities; etc. Idiopathic scoliosis is the most common form, accounting for approximately 75–85% of all scoliosis cases [1, 2]. Magnetic resonance imaging (MRI) is the gold standard for identifying idiopathic scoliosis, but the cause of scoliosis often remains unidentified due to the associated high costs and poor medical environments, e.g., long wait times and a lack of MRI equipment [3].

Intramedullary abnormalities can lead to scoliosis deformities, including Chiari malformations, syringomyelias, and hydromyelias [4]. Idiopathic scoliosis is difficult to diagnose based on a patient’s appearance. The gold standard MRI examination is not a routine scoliosis examination; therefore, patients with scoliosis caused by spinal cord abnormalities are easily misdiagnosed with idiopathic scoliosis, but there are differences in the treatment methods for these two forms of scoliosis [5]. The early treatment of idiopathic scoliosis is primarily supportive, and surgical correction may be required if the scoliosis continues to progress. Spinal scoliosis with intramedullary abnormalities should first be treated with neurosurgery to resolve the cause of scoliosis. Patients who are misdiagnosed and treated for idiopathic scoliosis who undergo direct scoliosis correction may suffer permanent nerve damage, leading to serious complications, such as lower limb weakness, pain and numbness, and even paralysis [6]. On the other hand, the early detection of scoliosis caused by intramedullary abnormalities and timely neurosurgical treatment can prevent further exacerbation; therefore, some scholars believe that MRI should be a routine examination for patients with scoliosis. However, other scholars believe that because the incidence of scoliosis caused by intramedullary lesions is low, MRI screening is a waste of money, time and medical resources; therefore, the diagnosis of scoliosis does not require an MRI examination, and an MRI examination can cause panic among patients, which is still the focus of debate [7].

In recent years, several articles have suggested that MRI examination of scoliosis patients is necessary. Studies have found that the incidence of scoliosis with spinal cord abnormalities is 6.3 to 9.9% [3, 4]. However, considering the current medical situation in China, it is particularly difficult for all patients with scoliosis to undergo an MRI examination of the whole spine. Therefore, this study aims to explore the risk factors for scoliosis caused by intramedullary abnormalities, identify the patient population that would benefit from selective MRI examination, explore the feasibility of MRI examination and provide a theoretical basis for the routine diagnosis and treatment of scoliosis.

## Method

The ethics committee approved the review of adolescent scoliosis surgery cases in Shanghai Tongren Hospital and Shanghai Changhai Hospital before December 2018 for this retrospective study.

### Research objective

1. Inclusion criteria: patients with scoliosis aged 10–18 years with complete spinal X-ray and MRI data.
2. Exclusion criteria: patients with congenital scoliosis (bone dysplasia), neuromuscular scoliosis (Patients with muscular dystrophy or mental retardation were excluded, while patients presenting only with a syringoid were not), neurofibromatosis scoliosis, metabolic scoliosis, Marfan syndrome and other clearly diagnosed scoliosis types.
3. Groupings: the spinal cord abnormality group (Group A) contained patients who exhibited lesions in the spinal cord, such as Chiari malformations, syringomyelia, and hydromyelia, on MRI examination. The idiopathic group (Group B) contained patients with no abnormalities on MRI examination.

### Data measurement and recording

**Fig. 1:**
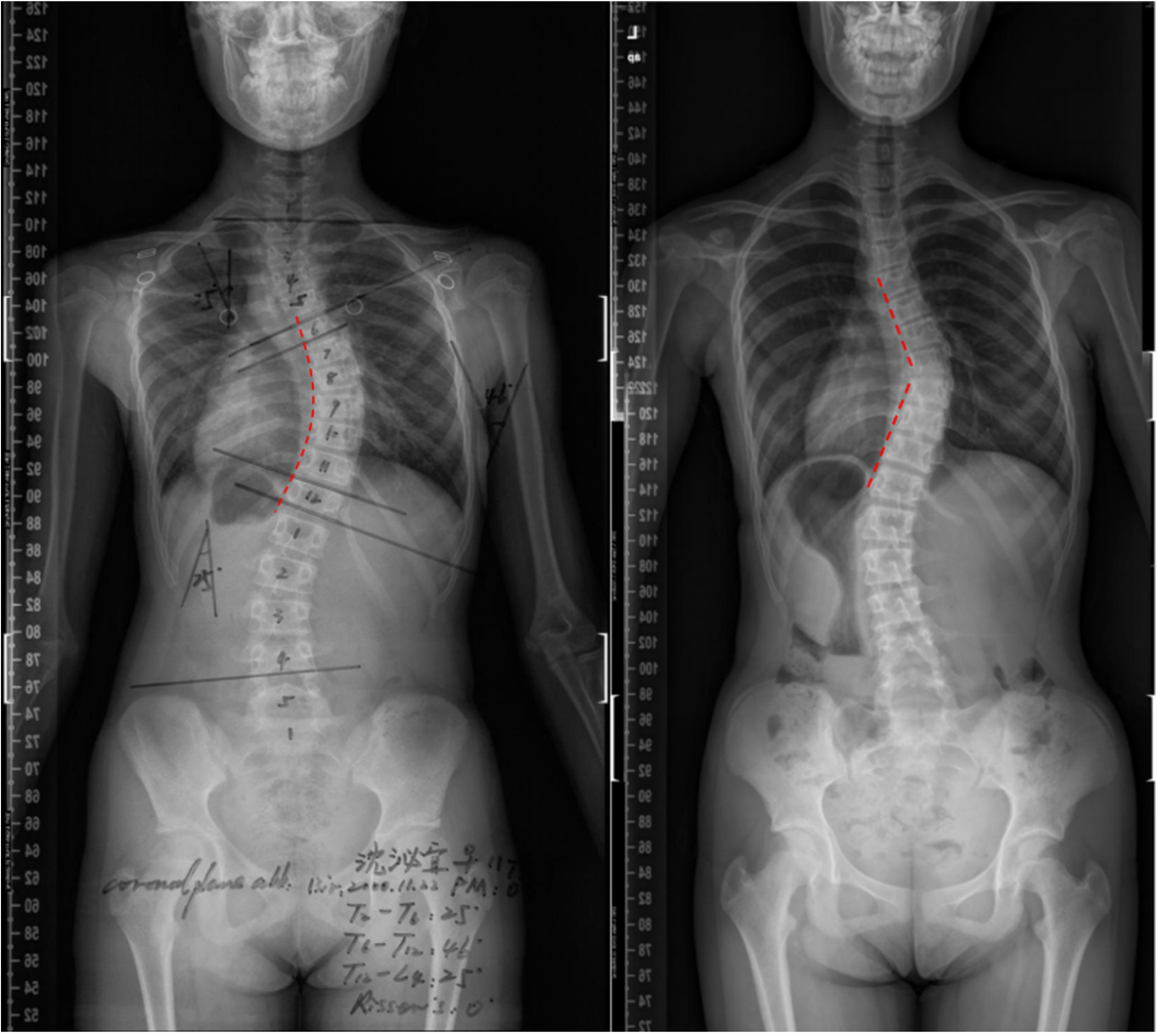
Two different scoliosis shapes

1. General information: age and sex.
2. Imaging examination: angle of the main bend, scoliosis direction (thoracic scoliosis on the left, thoracic scoliosis on the right), angle of thoracic vertebrae kyphosis, and scoliosis shape (Fig. 1, smooth shape is the concave side line is a smooth curve, angular shape is the concave side line is a angular curve).
3. Neurologic examination: assessment of motor, sensory, and reflex functions of the upper and lower extremities; pathologic signs; abdominal reflexes; tendon reflexes (radial membrane reflex, knee reflex, Achilles tendon reflex); paraesthesia; and ankle clonus.

### Clinical and imaging evaluation

A whole-spine 1.5 T Philips MRI machine (Philips, the Netherlands) was used to detect potential spinal abnormalities, including Chiari malformations, syringomyelia, vertebral compression, longitudinal spinal fractures, and spinal cord tumours. All diagnoses were made by a spine surgeon and reviewed by an experienced radiologist. According to the MRI findings, patients were assigned the spinal cord abnormality group or the idiopathic group to determine the imaging and clinical indicators of spinal cord abnormalities in the two groups.

Positive and lateral X-ray images of the whole spine were taken to measure the Cobb angle, scoliosis direction, scoliosis shape, thoracic kyphosis angle from T5 to T12 in the sagittal plane (defined as kyphosis deformity if the angle was greater than 10 degrees), the coronal plane balance (according to the central sacral vertical line (CSVL)), and the sagittal plane balance (according to the C7-S1 line) in the main bend. CSVL:A line passing through the midpoint of the upper edge of S1 perpendicular to the horizontal plane. C7-S1: The distance between the plumb line of C7 and the upper edge of S1.

### Statistical analysis

Sex, age, main curvature angle, kyphosis angle, scoliosis direction, scoliosis type, coronal plane balance, sagittal plane balance, abdominal wall reflex, sensory abnormalities, ankle clonus and tendon reflexes were compared between the spinal cord abnormality group and the idiopathic group. Statistical analyses were performed using the SPSS 21.0 statistical package (SPSS Inc., Chicago, IL). Continuous variables were compared using t tests, and rates were compared using chi-square tests. Fisher’s exact test was used to compare the rates of internal abnormalities between the groups. Logistic regression was used to evaluate the correlation between multiple variables and the incidence of intramedullary abnormalities, with the following values: intramedullary abnormalities =1 and no abnormalities =0. A *p* value less than 0.05 was defined as statistically significant.

## Results

Patient data from July 2013–December 2018 were retrieved from Tongren Hospital in Shanghai and from Shanghai Changhai Hospital. A total of 714 adolescent patients with scoliosis met the inclusion criteria. The patients had an average age of 13.5 (10 to 18) years, and 68 (9.5%) patients presented intramedullary abnormalities. Thirty-one patients underwent neurosurgical procedures, such as cerebellar tonsillar hernia resection, expanded foramen magnum decompression, vertebral reconstruction, and spinal cord cavity catheter drainage. The patients’ characteristics are shown in Table 1.

**Table 1:** Overview of spinal anomalies

Anomalies	Number of cases (%)
Isolated Arnold-Chiari malformation	49.5%
Arnold-Chiari malformation combined with syringomyelia	20.1%
Isolated syringomyelia	10%
Tethered cord combined with diastematomyelia	6%
Diastematomyelia	6%
Tethered cord	4%
Intrinsic spinal cord tumor	3%
Syringomyelia combined with tethered cord and tumor	1%
Total number	68

There were significant differences in the incidence rates of intramedullary abnormalities between males and females, patients with left and right thoracic curvatures, patients with angular scoliosis and smooth scoliosis, and patients with abdominal wall reflex abnormalities and ankle clonus. There were no significant differences between the other influencing factors, as shown in Table 2.

**Table 2:** Comparison between patients with and without neural abnormality on MRI screening examination

	Intramedullary Abnormalities *n* = 68	Idiopathic *n* = 646	*P*
Gender			
Male (1)	38	148	< 0.01^b^
Female(0)	30	498	
Age	14.1 ± 1.9	14.3 ± 1.7	NS^a^
Imaging			
Main Cobb	39.6 ± 8.1°	36.1 ± 11.7°	NS^a^
Left Thoracic curve (1)	15	74	0.012^b^
Right Thoracic curve(0)	53	572	
T-Kyphosis (1)	17	50	< 0.01^b^
No T-Kyphosis(0)	51	596	
Angular curve (1)	16	61	< 0.01^b^
Smooth curve(0)	52	585	
The trunk balance			
Coronal-imbalance (1)	5	42	NS^b^
Coronal-balance(0)	63	604	
Sagittal-imbalance (1)	9	70	NS^b^
Sagittal-balance(0)	59	576	
Nervous System			
Abnormal abdominal wall reflex (1)	19	40	< 0.01^b^
No-Abnormal abdominal wall reflex(0)	49	606	
Paresthesia (1)	10	52	0.064^b^
Euesthesia(0)	58	594	
Ankle clonus (1)	15	49	< 0.01^b^
No-Ankle clonus(0)	53	597	
Abnormal tendon reflex (1)	16	65	0.001^b^
Normal tendon reflex(0)	52	581	

NS indicates no statistical significance

^a^The student t test

^b^the chi-square test

Logistic regression showed that patients with intramedullary abnormalities were 2.987 times more likely to be male, were 3.493 times more likely to present scoliosis on the left thoracic side, were 4.823 times more likely to have lateral smooth scoliosis, were 3.94 times more likely to have abnormal abdominal wall reflexes, and were 8.083 times more likely to have ankle clonus than patients with idiopathic scoliosis, as shown in Table 3.

**Table 3:** Logistic regression results

	B	*P*	OR	95%CI
Gender	1.094	< 0.01		1.612–5.534
Female			1	
Male			2.987	
Direction of Scoliosis	1.251	< 0.01		1.756–6.948
R-Thoracic curve			1	
L-Thoracic curve			3.493	
T11-L2 Cobb	−0.974	0.054		0.140–1.016
Normal			1	
Kyphosis			0.377	
Shape of Curve	1.573	< 0.01		2.278–10.211
Smooth curve			1	
Angular curve			4.823	
Coronal Plane	−0.858	0.112		0.147–1.223
Imbalance			1	
Balance			0.424	
Sagittal Plane	−0.226	0.637		0.312–2.041
Imbalance			1	
Balance			0.798	
Abdominal reflexes	1.371	< 0.01		1.810–8.574
Normal			1	
Abnormal			3.940	
Feeling	−0.119	0.817		0.324–2.433
Normal			1	
Abnormal			0.888	
Ankle Clonus	2.090	< 0.01		3.945–16.562
Normal			1	
Abnormal			8.083	
Tendon Reflex	−0.828	0.088		0.168–1.132
Normal			1	
Abnormal			0.437	
Constant	−3.522	< 0.01	0.030	

Regression equation:

$$ \mathrm{Logit}\ \left(\mathrm{P}\right)=-3.522+1.094\ \mathrm{sex}+1.251\ \mathrm{scoliosis}\ \mathrm{direction}+1.573\ \mathrm{scoliosis}\ \mathrm{shape}+1.371\ \mathrm{abdominal}\ \mathrm{wall}\ \mathrm{reflex}+2.090\ \mathrm{ankle}\ \mathrm{clonus} $$

**Fig. 2:**
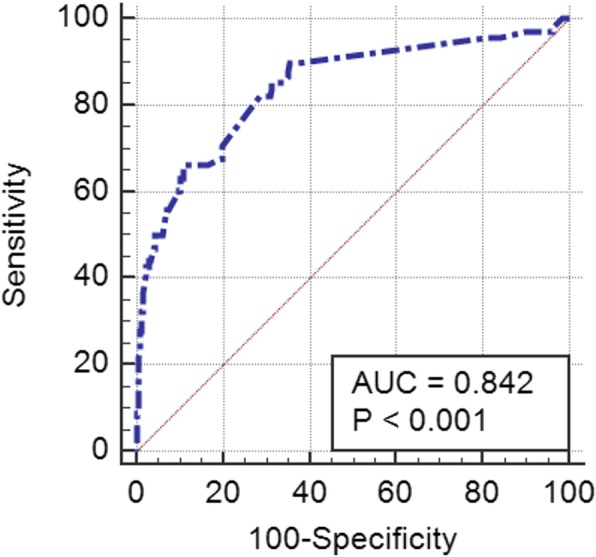
ROC curve for predicting intramedullary abnormalities

**Fig. 3:**
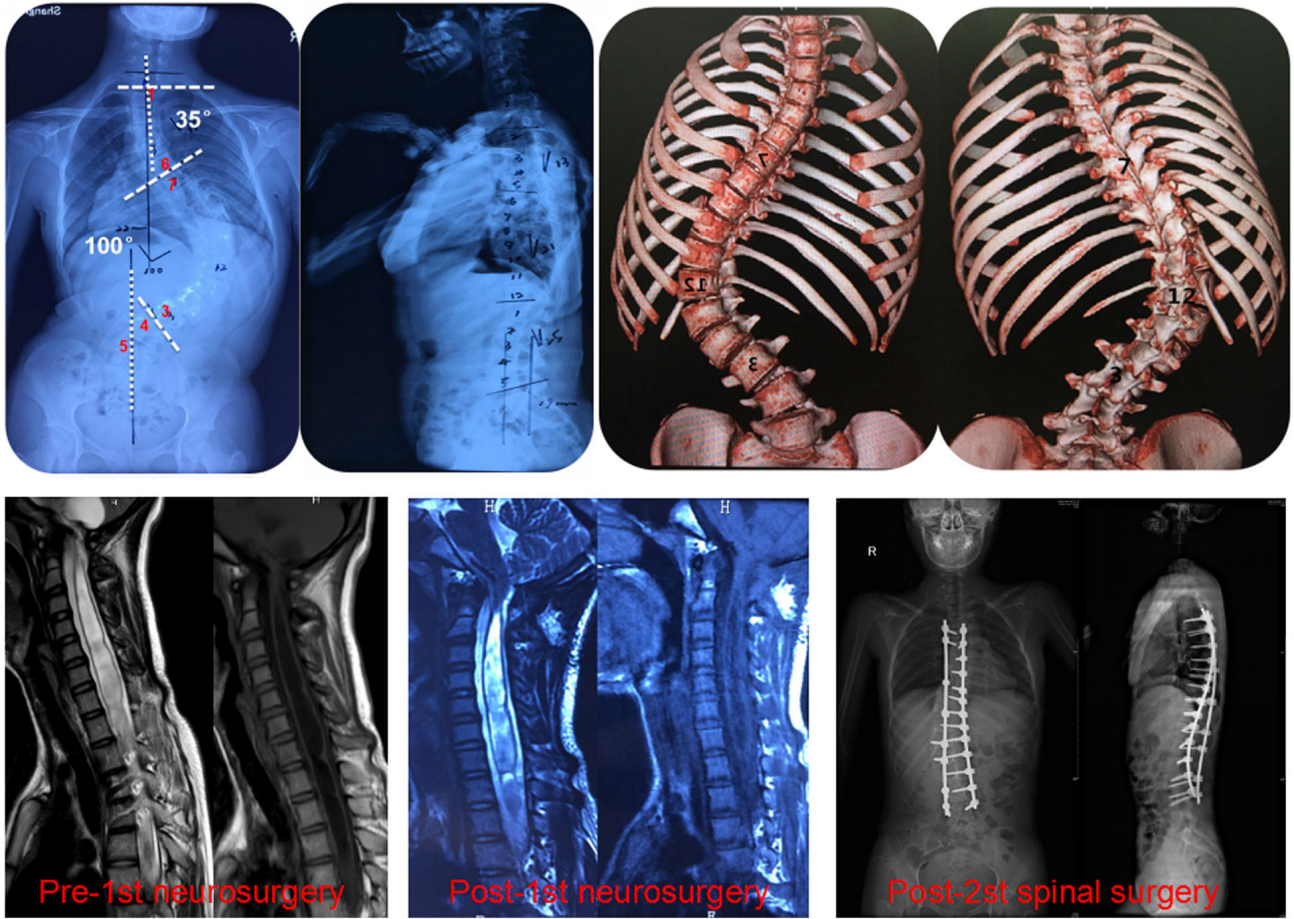
Typical case

The area under the receiver operating characteristic (Receiver Operating Characteristic, ROC) curve for the incidence of intramedullary abnormalities was 0.842 (95% confidence interval: 0.813–0.868, *P* < 0.001). The sensitivity was 66.18%, the specificity was 89.01%, and the Youden index corresponding to the optimal critical point was 0.5519, as shown in Fig. 2. The ROC reflects the relationship between the sensitivity and specificity of the prediction formula. Generally speaking, an AUC (Area Under Curve) between 0.7 and 0.9 indicates high accuracy of the prediction formula. Figure 3 shows a 13-year-old female who weighed 38 kg, was 138 cm tall and had a spinal deformity for 1 year.

Figure 3 NOTE: Coronal Plane: T1-T6: 35°, T7-L3:100°, CB: 22 mm, Risser: 0~I°

Sagittal Plane: T2-T5: 13°, T5-L12: 21°, L1-L5: 25°, SVA: 19 mm

The patient presented left thoracic curvature, right lumbar curvature, an abnormal abdominal wall reflex, paraesthesia, and ankle clonus.

The whole-spine MRI showed a Chiari malformation and syringomyelia.

First step: In the first step of the patient’s treatment, Neurosurgery department performed posterior fossa decompression. Second step: In the second step, Spinal surgery was performed to correct scoliosis from T4 to L4.Spinal surgery was performed 3–6 months after neurosurgery.

## Discussion

According to the appointment registration statistics of major hospitals in Shanghai, the wait time for a spinal MRI appointment is approximately 4 weeks, and the cost of an MRI examination for a single spinal section (cervical spine, thoracic spine, and lumbar spine) is 460 RMB (65 USD). A whole-spine MRI examination costs 1380 RMB (196 USD); however, 90% of the cost will be reimbursed by Shanghai Medical Insurance, and only 10% will be paid by the patients themselves. So the MRI is still a cost-effective test and I want to call on the patient to have an MRI. There were a lot of severe scoliosis due to no early diagnosis, and I thought if we could find it early by MRI, we could treat it early.

According to previous studies and this study, the incidence of intramedullary abnormalities in patients initially diagnosed with idiopathic scoliosis is approximately 6.3 to 9.9% [4]. As 90.1 to 93.7% of patients have idiopathic scoliosis, a whole-spine MRI to determine whether there is an intramedullary abnormality could be considered a waste of resources. Therefore, some scholars believe that whole-spine MRI is not necessary for idiopathic scoliosis patients [8]. However, there have been cases of spinal cord injury in patients with idiopathic scoliosis who underwent surgical correction, with very serious consequences [9]. The purpose of this study was to develop accurate intramedullary abnormality screening criteria to reduce medical resource waste. A retrospective study with a large sample size found that intramedullary abnormalities were associated with five risk factors. The results showed that if the incidence of the five factors associated with intramedullary abnormality was 66.18%, an MRI should not be conducted, but if the incidence rate of the five factors was 89.01%, the patient should undergo a whole-spine MRI. Therefore, regarding selective whole-spine MRI, applying the five risk factors as a condition for whole-spine MRI can improve the whole-spine MRI intramedullary abnormality positivity rate and reduce unnecessary medical treatments. This scheme is completely feasible in the current Chinese medical environment. The more consistent the risk factors are, the higher the probability of intramedullary abnormalities will be. Selective examinations can be conducted for high-risk patients to further clarify the aetiology of adolescent scoliosis, which provides a theoretical basis for selective MRI examination and has important clinical practical significance.

There are not many relevant studies searched on pubmed. Choon et al. [9] suggested that males and patients with increased thoracic kyphosis are risk factor. Zhang et al. [10] considered that aged less than 10 years, being male or having left thoracic or right lumbar curve are risk factor. Beyond that long curve span, apex at thoracolumbar spine and hyperthoracic kyphosis and so on are also risk factor [3, 6–8, 10–25]. This study includes the second largest research sample to date. The risk factors identified in this study can be divided into three categories, namely, general, imaging, and neurologic characteristics; a total of 12 clinical indicators were included in the correlation analysis. The content of this study is more comprehensive than that in previous studies.

This study retrospectively analysed the incidence of intramedullary abnormalities in adolescent scoliosis patients and proposed the idea of selective whole-spine MRI examination, which has a strong guiding role in clinical work. However, this study had the following limitations. 1. Patients from two hospitals were included in the analysis, and this study contains the second largest sample size (714 patients) in the current literature. However, the sample size is still insufficient to study disease incidence, although this did not affect the research conclusion. 2. Although 12 factors were included in this study, the specificity (66.18%) and sensitivity (89.01%) for predicting intramedullary abnormalities still require further improvement; the factors affecting intramedullary abnormalities included in this study are not sufficiently comprehensive. 3. Due to the limitations of retrospective studies, the data must be supplemented with additional prospective studies that include further analyses of influencing factors and larger sample sizes to obtain more-conclusive research results.

In this study, the incidence of adolescent scoliosis caused by an intramedullary abnormality was 9.5%. MRI assessment showed that the spinal cords of patients were not excessively damaged. We recommend that high-risk patients (males and those with a lateral thoracic spine angle on the left side of the curve, an angular curve, abdominal wall reflection and ankle clonus) undergo selective spinal MRI, because neurological complications (paraplegia, nerve damage, etc.) can cause irreparable damage. These complications can be prevented by preoperative management. For example, syringomyelia causes scoliosis, and scoliosis correction before appropriate neurosurgery or surgical interventions can avoid spinal cord damage during scoliosis correction surgery. Therefore, the spine surgeon should pay close attention to the diagnosis of preoperative patients. Currently, we are using this method to conduct a prospective study on high-risk adolescent patients with scoliosis to further verify the feasibility of this method and further identify risk factors while increasing the sample size to further improve the prediction equation.

## Conclusion

Adolescent scoliosis caused by intramedullary abnormalities is associated with male sex, thoracic scoliosis on the left side, a sharp curve, abdominal wall reflection and ankle clonus. The incidence of adolescent scoliosis caused by intramedullary abnormalities was approximately 9.5%. Clinical indicators suggest that there is a high-risk population of adolescent patients with scoliosis who should undergo whole-spine MRI preoperatively to rule out intramedullary abnormalities.

## Data Availability

Not applicable.

## Abbreviations

AUC: Area Under Curve
CSVL: Central Sacral Vertical line
MRI: Magnetic Resonance Imaging
ROC: Receiver Operating Characteristic
